# Beyond Reporting: Claude 3.7 Sonnet Accurately Classifies T Stage and Uncovers Omitted Anatomic Invasion in Nasopharyngeal Carcinoma Magnetic Resonance Imaging (MRI) Reports

**DOI:** 10.7759/cureus.91209

**Published:** 2025-08-28

**Authors:** Yusuke Asari, Ryo Kurokawa, Akifumi Hagiwara, Mariko Kurokawa, Yuki Sonoda, Jun Kanzawa, Shota Fujisawa, Sousuke Hatano, Wataru Gonoi, Osamu Abe

**Affiliations:** 1 Radiology, The University of Tokyo, Tokyo, JPN; 2 Radiology, Juntendo University, Tokyo, JPN

**Keywords:** artificial intelligence, claude 3.7 sonnet, large language models, magnetic resonance imaging, nasopharyngeal carcinoma, tnm classification

## Abstract

Purpose

This study aimed to determine whether Claude 3.7 Sonnet (Anthropic, San Francisco, CA, USA), a large language model (LLM), can (i) assign nasopharyngeal carcinoma (NPC) T classification from routine magnetic resonance imaging (MRI) reports and (ii) identify unreported anatomical structures whose invasion would warrant a higher T stage.

Materials and methods

This single-institution retrospective study included 38 consecutive patients (31 men; mean age 59.7±14.9 years) who underwent pretreatment MRI for NPC between April 1999 and March 2025. De-identified unstructured "Findings" sections were submitted once to Claude 3.7 Sonnet (temperature=0), prompting the model to assign a T stage according to the American Joint Committee on Cancer/Union for International Cancer Control 9th Edition and to list potentially missed invasive sites. Reference-standard staging and relevant omissions were established independently by two radiologists. Model accuracy for T classification and for detecting missing structures was calculated; false-positive flags were recorded. Radiologists re-evaluated MR images for any stage change prompted by the LLM.

Results

The LLM reproduced the reference T category in 35/38 patients (92.1%). Category-specific accuracy was 100% for T1 (9/9) and T4 (13/13), 90% for T3 (9/10), and 66.7% for T2 (4/6). Among 208 eligible unmentioned structures, the model correctly flagged 81 (38.9%), with a mean of 3.34 false-positive suggestions per case. Subsequent human review confirmed stage upgrades in 2/38 patients (5.3%), both corrected to T4 based on intracranial extension or cranial nerve involvement noted by the LLM.

Conclusion

Claude 3.7 Sonnet achieved high accuracy in T staging from unstructured free-text MRI reports for NPC and identified clinically important omissions, enabling radiologists to correct staging in select cases. LLM-assisted report auditing may improve staging quality and serve as an educational aid where subspecialty expertise is limited.

## Introduction

Nasopharyngeal carcinoma (NPC) is a malignancy arising from the nasopharyngeal epithelium. NPC exhibits striking geographical disparities, with age-standardized incidence rates reaching 20-30 per 100,000 in Southern China and Southeast Asia, and in Epstein-Barr virus (EBV)-associated cases, particularly the non-keratinizing subtype, tumor cells harbor clonal EBV episomes [1,2]. The male-to-female incidence ratio remains consistently elevated at 2-3:1 across endemic populations. EBV infection is ubiquitous in tumor cells, with viral deoxyribonucleic acid (DNA) detectable in 96% of non-keratinizing NPC cases [1,2]. Host genetic susceptibility, such as HLA-A*02:07 variants, acts synergistically with environmental factors, including the consumption of salted fish, to drive carcinogenesis [1,2].

The 9th Version of the American Joint Committee on Cancer/Union for International Cancer Control (AJCC/UICC) tumor, node, and metastasis (TNM) classification for NPC was recently introduced, by incorporating more detailed anatomical criteria to improve staging accuracy and prognostic stratification. The 9th Version retains the same overall T classification structure as the 8th Version and emphasizes specific anatomic spread (e.g., involvement of prevertebral muscles for T2 and cervical vertebrae for T3) that defines advanced disease [3]. Accurate cancer staging at diagnosis, especially T classification, is critical for disease management and outcome prediction [3,4]. Given the complex anatomy of the nasopharynx and surrounding regions, imaging plays a central role in NPC T staging. In particular, magnetic resonance imaging (MRI) is the preferred modality for evaluating the extent of the primary tumor. MRI has superior soft-tissue contrast and sensitivity for detecting deep tumor invasion compared to computed tomography (CT) [5]. 

In recent years, artificial intelligence (AI) techniques have been investigated to assist radiological interpretation. Large language models (LLMs) such as OpenAI's GPT-4 and GPT-4o (San Francisco, CA, USA) and Anthropic's Claude 3.5 Sonnet (San Francisco, CA, USA) have drawn particular interest for their ability to understand context and generate human-like text [6]. Such models, trained on vast amounts of data, have demonstrated impressive results on medical knowledge tasks, for instance, LLMs have achieved high scores on the national medical licensing exam and the radiology board certification exam [7-9]. In the field of radiology, LLMs have shown strong performance in diagnostic quiz cases, and comparisons of their performance, as well as studies on optimal prompting strategies, are currently ongoing [10-14]. LLMs hold promise for processing textual data, such as radiology reports, to extract clinically useful information [15-17]. Early studies have demonstrated their ability to interpret free-text radiology reports and accurately output cancer staging and resectability information [18-20]. These findings suggest that LLMs can effectively capture key information from radiology reports for staging purposes, particularly for clear-cut findings such as metastatic disease.

General radiologists who are not head and neck specialists may struggle with nuanced interpretations of NPC cases, which can result in staging errors or ambiguities in reports. Prior research on second-opinion reviews underscores the limitations of non-expert initial interpretations [21]. This indicates that important anatomic findings are sometimes missed or underreported by non-specialists, leading to understaging or overstaging. NPC T staging, in particular, requires meticulous attention to subtle areas of invasion that a general radiologist might overlook in a busy practice. This gap presents an opportunity for AI assistance: a system that can read a routine MRI report and deduce the T stage, and even identify if a key structure is not mentioned, would be highly valuable in ensuring the completeness and accuracy of staging.

This study aims to evaluate the ability of LLMs to accurately determine the T stage of NPC and identify omissions of key anatomical information in MRI reports.

## Materials and methods

Study design and ethical considerations

**Figure 1:**
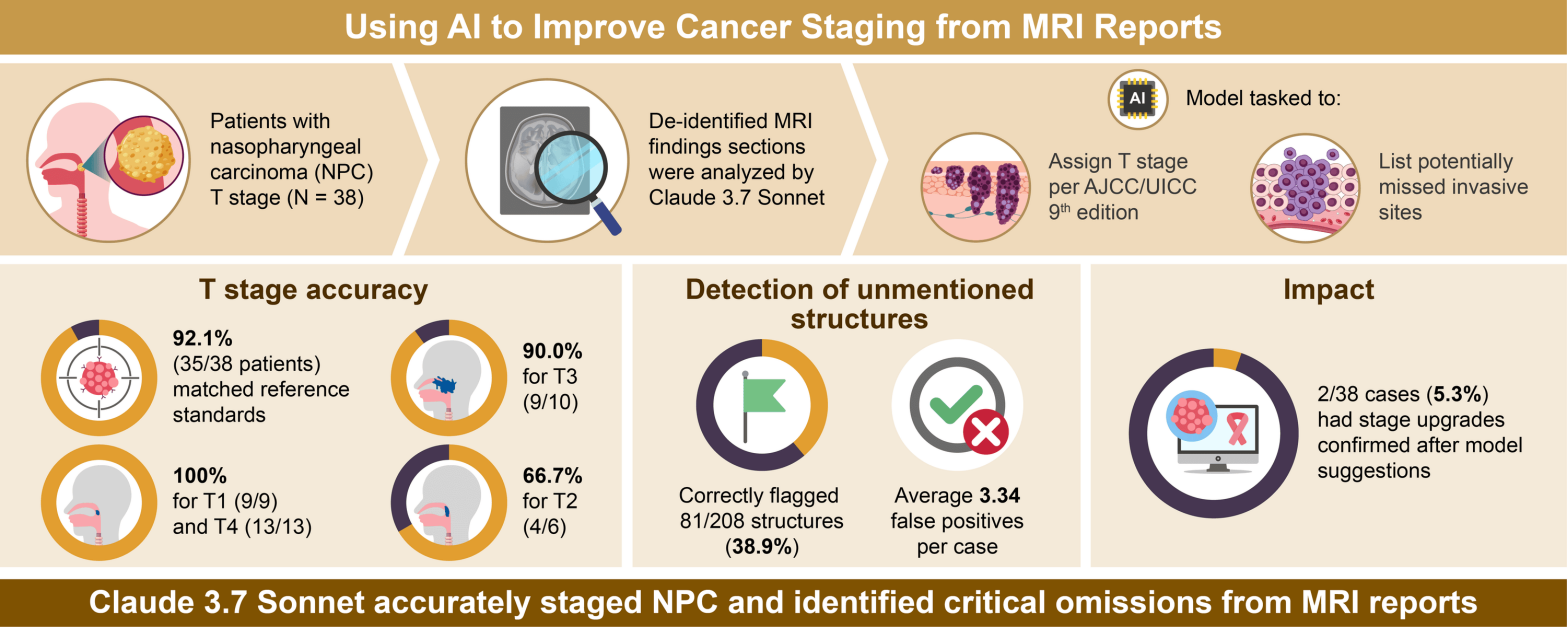
Overview of the study NPC: nasopharyngeal carcinoma; AJCC/UICC: American Joint Committee on Cancer/Union for International Cancer Control; AI: artificial intelligence; MRI: magnetic resonance imaging Image Credit: Authors

This single-institution retrospective study was conducted in accordance with the principles of the Declaration of Helsinki and was approved by the Research Ethics Committee of the Graduate School of Medicine and Faculty of Medicine of the University of Tokyo (approval number: 2024425NI-(1)). The need for individual informed consent was waived due to the retrospective design of the study and the use of anonymized data. Personal identifiers of all MRI reports were removed before analysis. The overview of the study is summarized in Figure 1.

Patients and data collection

We retrospectively identified 38 consecutive patients who underwent preoperative MRI for newly diagnosed NPC at the University of Tokyo, Japan, between April 1999 and March 2025. No exclusion criteria were applied. All reports were written in Japanese, and only the "Findings" section of the reports was used in subsequent analysis.

Prompting strategy

We used Claude 3.7 Sonnet and application programming interfaces (API) to access the model (Claude 3.7 Sonnet: claude-3-7-sonnet-20250219) on April 8, 2025. All analyses were conducted using the Claude 3.7 Sonnet model of the same version within a consistent API environment. This specific model was selected due to its distinguished performance in quiz-based clinical reasoning tasks in the field of diagnostic radiology, as evidenced by recent comparative studies [10,11]. To ensure reproducibility, the generation parameters for all models were set to a temperature of 0.0. Each prompt was submitted in an independent session to prevent previous inputs from influencing subsequent ones. The prompt was written in Japanese as follows:

You are a specialist in head and neck tumors. Based on the following MRI interpretation report, determine the T classification according to the AJCC & UICC 9th edition TNM classification for head and neck cancer. Please assume that there is no invasion into any anatomical structures that are not mentioned in the report. MRI Report:{report_text}. Please respond in JSON format, specifying the T classification (e.g., "T1", "T2", "T3", "T4"), listing the anatomical structures whose invasion is not mentioned in the report but could potentially lead to a higher T classification, and for each of these structures indicating the potential T classification if invasion is confirmed.

We ensured that both the prompt and the report text were written in Japanese to minimize potential bias that could be caused by translation and interpretation. We submitted each prompt to the model only once and used the first generated response for evaluation. 

Output analysis

One board-certified radiologist with 12 years of head and neck imaging experience and one radiology resident with four years of head and neck imaging experience independently reviewed the reports and assigned a reference-standard T classification and identified missing anatomical elements for each case using the 9th Version of AJCC/UICC TNM classification. Any disagreement was resolved by consensus. The accuracy of the T classification generated by the LLM was then calculated. 

Next, the accuracy of identifying missing anatomical structures was analyzed. According to the 9th Version of the AJCC/UICC TNM classification, the anatomical structures required to determine the T classification are as follows: for T2, the parapharyngeal space, medial pterygoid muscle, lateral pterygoid muscle, and prevertebral muscle; for T3, the skull base (including pterygoid structures), paranasal sinuses, and cervical vertebrae; and for T4, the intracranial extension, cranial nerves, hypopharynx, orbit (including the inferior orbital fissure), parotid gland, and extensive soft-tissue infiltration beyond the anterolateral surface of the lateral pterygoid muscle.

For each case classified as T1, T2, or T3, we counted the number of anatomical structures that could upgrade the T classification (out of 13 items for T1, nine items for T2, and six items for T3) that were not mentioned in the reports but flagged by the LLM. The output was considered correct if the LLM accurately identified both the structure name and the potential upgraded T classification. We defined any structure flagged by the LLM that was either mentioned in the report or irrelevant to upgrading the T classification as a false positive. 

The two radiologists reviewed each actual MR image and checked whether the unmentioned structures flagged by the LLM might warrant an upgrade in the T classification. For cases where T staging was upgraded upon re-evaluation by radiologists, we examined whether the upgrade should have occurred according to the T classification edition in use at the time of the report (whether the T stage was underestimated at the time of report creation).

## Results

Patient characteristics

The study population consisted of 31 men and seven women, with a mean age of 59.7±14.9 years. Intravenous contrast medium was utilized in 37 of 38 cases (97.4%). T classification based on radiology reports was as follows: T1 (n=9), T2 (n=6), T3 (n=10), and T4 (n=13).

LLM-based T classification accuracy

Among the 38 patients, the LLM accurately predicted the correct T stage in 35 cases (92.1%, Table 1).

**Table 1:** Accuracy of LLM-based T classification and detection of unmentioned anatomical structures LLM: large language model

T category	Number of cases	T classification accuracy	Correctly flagged structures	False-positive flags per case
All T	38	92.1% (35/38)	38.9% (81/208)	3.34
T1	9	100% (9/9)	40% (42/105)	2.00
T2	6	66.7% (4/6)	35.4% (17/48)	3.67
T3	10	90% (9/10)	40% (22/55)	3.20
T4	13	100% (13/13)	NA	4.23

The three misclassifications consisted of the following: one T2 tumor misclassified as T3, one T2 tumor misclassified as T4, and one T3 tumor misclassified as T4.

Missing Anatomical Structures and Accuracy of LLM Suggestions

Across the 38 MRI reports, the LLM correctly flagged 81 of 208 anatomical structures that were unmentioned but would have led to a higher T classification if infiltration had been present, resulting in a recall of 38.9%. Notably, the total number of structures flagged by the LLM (208) coincidentally matched the number of structures that should have been flagged (208), although they were determined independently. Of the 208 total flags generated by the LLM, 81 were judged correct upon review, yielding a precision of 38.9%. The remaining 127 were considered false positives. On average, the LLM produced 3.34 false-positive flags per case (Table 1). 

The 127 false-positive flags can be categorized as follows: 57 cases involved anatomical structures described under the same T category or a lower T category (e.g., recommending paranasal sinus checks for T4 cases), 55 cases described anatomical structures not listed in the AJCC/UICC classification (e.g., skin infiltration or carotid artery infiltration), nine cases involved incorrect T classifications, and six cases involved anatomical structures already mentioned in the report.

Changes to T stage following LLM review

**Figure 2:**
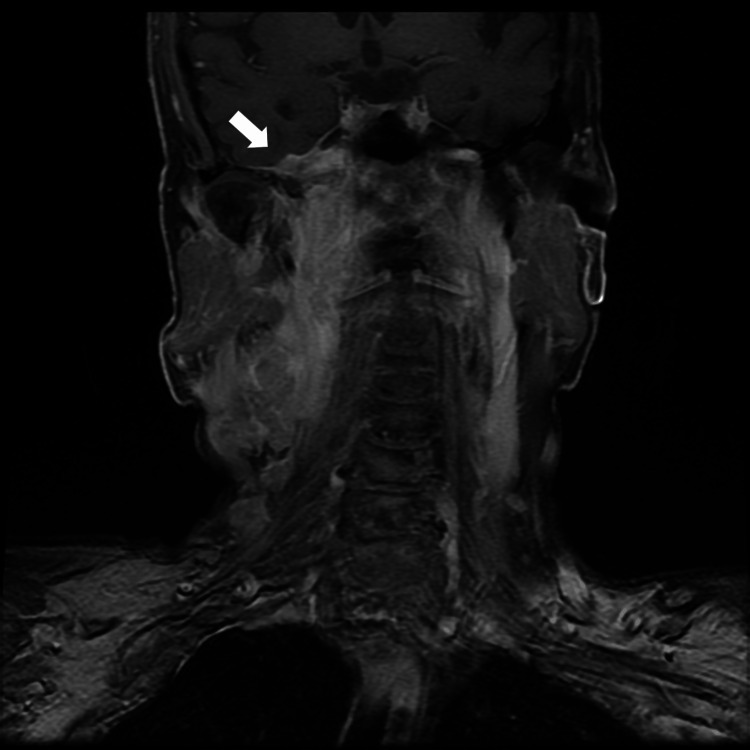
Post-contrast T1-weighted coronal MR image of nasopharyngeal carcinoma in a 67-year-old male patient The case was initially reported as T3; however, a final classification of T4 was made based on the LLM flag indicating intracranial extension. The arrow indicates the area of intracranial extension. LLM: large language model

**Figure 3:**
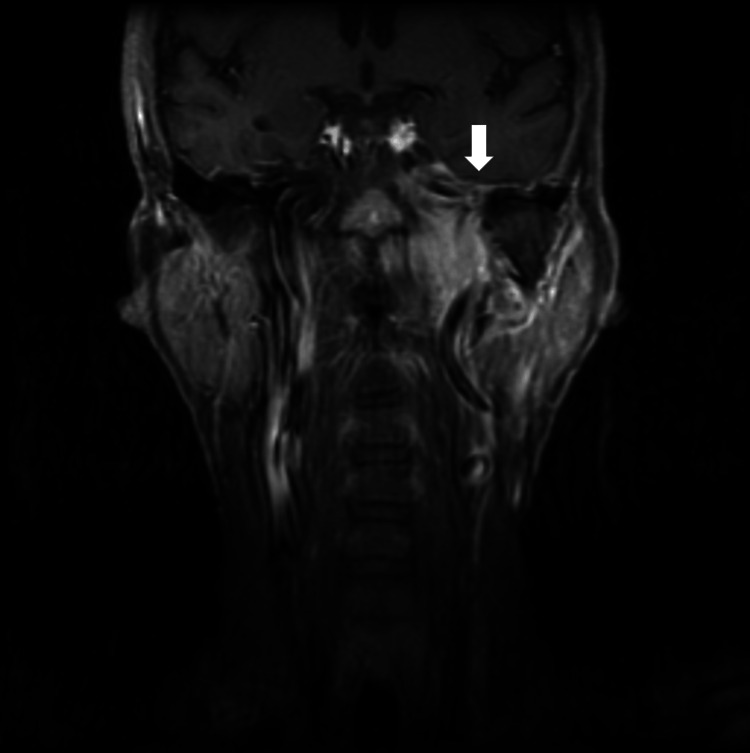
Post-contrast T1-weighted coronal MR image of nasopharyngeal carcinoma in a 59-year-old male patient The case was initially reported as T3; however, a final classification of T4 was made based on the LLM flag indicating cranial nerve involvement. The arrow indicates the area of cranial nerve involvement. LLM: large language model

In two out of 38 cases, the T classification was corrected based on anatomical structures flagged by the LLM. Specifically, one case had intracranial extension and the other case had cranial nerve involvement, leading to a final decision of T4 instead of the originally reported T3 (Figure 2 and Figure 3).

These upgrades would have also applied under previous editions of the AJCC classifications in use at the time the original reports were written.

## Discussion

In this study, we found that Claude 3.7 Sonnet accurately predicted the T stage of NPC in 92.1% of cases (35 out of 38) based on MRI reports. Beyond replicating the T category, we found that the LLM correctly identified 38.9% of anatomical structures, which had not been previously documented but were indeed relevant for upgrading the T classification. Moreover, in two of 38 cases, human radiologists were able to revise the T classification after considering the LLM's suggestions. To our knowledge, this is the first documented instance where an LLM's output prompted radiologists to modify their staging assessment in real clinical cases.

The need for general radiologists to interpret images in areas where they may lack subspecialty expertise is a common challenge. More than half (55.3%) of radiologists primarily practice as general radiologists in the United States, very few institutions are organized by subspecialties in Latin America, and only 11% of radiologists exclusively interpret images within their specific subspecialty area in Japan [22,23]. In clinical settings, subspecialty expertise is not always available for every radiological interpretation. By providing accurate T staging based on the latest classification systems and reminding human radiologists of critical anatomical structures not mentioned in radiology reports, LLMs can effectively complement radiological reporting. This capability offers valuable support for radiologists who are not specialized in specific anatomical domains, as well as for trainee radiologists. Simultaneously, it functions as an educational resource, offering real-time, case-based feedback during clinical practice.

A prior investigation into the application of LLMs for TNM classification reported a 47% accuracy rate for T classification using GPT-3.5 Turbo on English lung cancer CT reports [18]. Other research showed that GPT-4o achieved an overall lung cancer staging accuracy of 74.1% when analyzing CT and fluorodeoxyglucose positron emission tomography/CT (FDG PET/CT) reports [24]. In another study focused on pancreatic cancer, GPT-4 paired with chain-of-thought prompting demonstrated high accuracy in assessing tumor resectability from CT reports [19]. While these studies have demonstrated the ability of LLMs to accurately perform cancer staging based on reports created by human radiologists, efforts to further improve the accuracy of these reports have not been undertaken. Our research has newly demonstrated that in addition to the high accuracy of T staging based on human radiologists' reports, LLMs could appropriately identify anatomical structures that were insufficiently mentioned in the original reports, and furthermore, this led to actual corrections in T staging. The present study exemplifies the dual role of LLMs not only as diagnostic support tools but also as educational aids, an area of growing interest in medical AI applications [16]. 

Despite the promising performance, we must acknowledge the risks of LLM outputs in this context. On average, there were 3.34 incorrect structure flags per case. These false-positive suggestions reflect the well-known tendency of LLMs to "hallucinate" plausible but inaccurate information [25]. Therefore, staging or findings proposed by the AI must be verified by a radiologist or a multidisciplinary team before being used to influence patient management. 

Several limitations of our study must be considered. First, this was a single-center retrospective study with a relatively small sample size, which limits the generalizability of our findings to broader clinical settings. Second, the MRI acquisition protocols were continuously updated, resulting in some inconsistency across patients. Third, although we evaluated the LLM using existing MRI reports, these were authored by multiple radiologists with varying levels of expertise and reporting styles. This inter-radiologist variability could have influenced both the baseline completeness of the reports and the model's performance. Fourth, while the model flagged an average of 3.34 anatomical structures per case, this relatively high number of prompts may have implications for workflow efficiency and could contribute to alert fatigue in clinical environments. Fifth, the model's performance is tied to a specific proprietary LLM, which may affect reproducibility if access or versions change over time. Generalizability to other language models or institutions using different infrastructure remains uncertain. Finally, this study did not include prospective clinical validation. While retrospective evaluation allows for controlled assessment, it does not replicate real-world conditions. Future studies should assess the performance and usability of LLMs in real-time reporting environments to determine whether the improvements observed here translate to clinical practice.

## Conclusions

Our study provides preliminary evidence that Claude 3.7 Sonnet may achieve accurate T staging of NPC based on human radiologists' MRI reports and has the potential to identify omissions in those reports. While these findings highlight a promising step toward LLM-assisted radiology reporting in cancer staging, the results should be interpreted with caution due to the small, single-center sample, the absence of a control group, and a relatively high false-positive rate for flagged structures. Further prospective, multi-center validation is necessary before this technology can be considered ready for widespread clinical adoption. Nonetheless, this study underscores the potential of such tools as decision support and educational aids for radiologists.
